# Exploring new natural products by utilizing untapped secondary metabolic pathways in actinomycetes

**DOI:** 10.1007/s11418-025-01903-9

**Published:** 2025-04-04

**Authors:** Shotaro Hoshino

**Affiliations:** Department of Life Science, Faculty of Science, Gakushuin University, 1-5-1 Mejiro, Toshima-ku, Tokyo, 171-8588 Japan

**Keywords:** Actinomycetes, Biosynthesis, Combined-culture, Mycolic acid-containing bacteria (MACB), Organoarsenic natural product, Secondary metabolites

## Abstract

Actinomycetes have produced a variety of bioactive secondary metabolites; however, discovering new actinobacterial natural products using conventional approaches has become increasingly challenging. Meanwhile, genomic studies of actinomycetes have revealed that numerous secondary metabolite biosynthetic gene clusters (SM-BGCs) remain untapped. Thus, utilizing these secondary metabolic pathways is expected to facilitate the discovery of new actinomycetes-derived natural products. In this review, I primarily describe our research on the utilization of these untapped actinobacterial SM-BGCs and the discovery of new secondary metabolites. First, I introduce our studies on the activation of silent SM-BGCs through the co-cultivation of various actinomycetes with mycolic acid-containing bacteria (MACB), which led to the identification of 20 actinobacterial secondary metabolites, including 16 new compounds. In the latter part, I describe our recent findings on arsenic-related secondary metabolism, which has been overlooked in model actinomycetes, including the identification of a novel organoarsenic natural product, and the elucidation of its unique biosynthetic strategy, which is independent of *S*-adenosylmethionine (SAM)-dependent enzymes.

**Graphical abstract:**

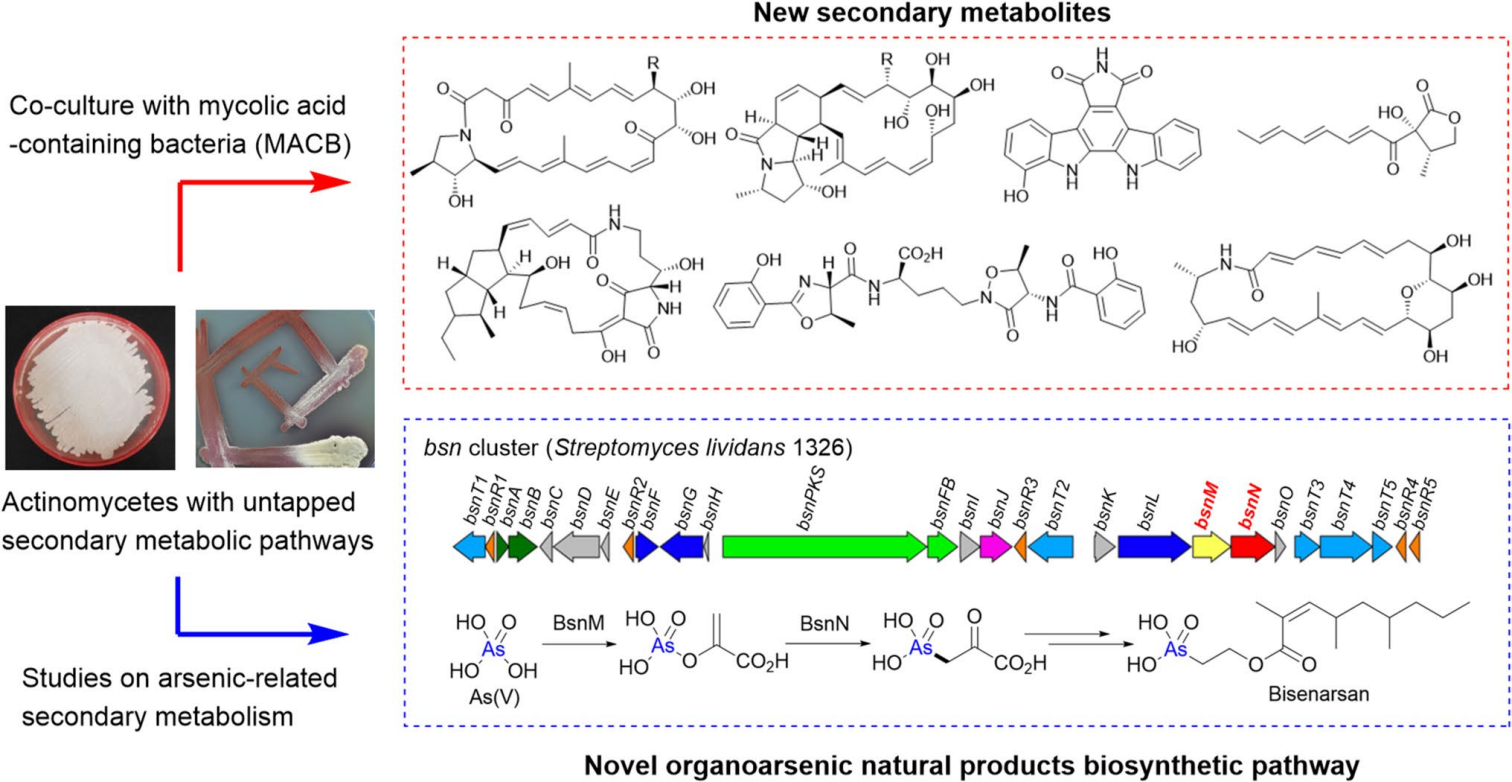

## Introduction

Bacterial secondary metabolites often exhibit remarkable bioactivities and have significantly contributed to human society through their application in pharmaceuticals, agrochemicals and food additives. In particular, since the discovery of actinomycins and streptomycin [1, 2], numerous bioactive secondary metabolites have been identified from actinomycetes [3–5]. However, the continuous exploration of actinomycetes has made the discovery of new natural products increasingly challenging.

Meanwhile, genomic studies have revealed that actinomycetes harbor numerous secondary metabolite biosynthetic gene clusters (SM-BGCs) [6–8], far exceeding the total number of actinobacterial secondary metabolites identified to date. Thus, the majority of SM-BGCs remain cryptic under standard culture conditions, and activating these untapped actinobacterial SM-BGCs is expected to facilitate the discovery of new bioactive compounds. In fact, new secondary metabolites have been successfully identified through the activation of cryptic SM-BGCs using heterologous expression [9, 10], the application of chemical elicitors [11, 12], and genetic manipulation [13].

Furthermore, secondary metabolism involving elements that are rarely found in natural products is also noteworthy. For example, some actinomycetes possess specific SM-BGCs and produce organofluorine [14–16] and organoselenium metabolites [17], representing an extremely rare class of natural products. However, a large portion of these SM-BGCs is likely overlooked, even if they are functional, because conventional culture media typically lack the necessary elements for their biosynthesis.

Under these circumstances, we have continued our efforts to discover new natural products by utilizing untapped actinobacterial SM-BGCs. In this review, we first describe our studies on the activation of actinobacterial SM-BGCs through co-culture with mycolic acid-containing bacteria (MACB). In the latter part, we present our recent findings on actinobacterial secondary metabolism related to organoarsenical natural products.

### Combined-culture: effective activation of actinobacterial SM-BGCs through co-culture of actinomycetes with MACB

Co-culture is also an effective approach for activating cryptic SM-BGCs, and several co-culture studies have successfully led to the isolation of new microbial natural products [18–20]. It is a simple and broadly applicable method that does not require genetic manipulation or expensive reagents. However, conventional co-culture approaches often require extensive screening to identify suitable microbial combinations. To address this limitation, Onaka et al. proposed an efficient co-culture strategy using MACB as a partner to activate cryptic SM-BGCs in actinomycetes, which they termed "combined-culture" [21].

**Fig. 1:**
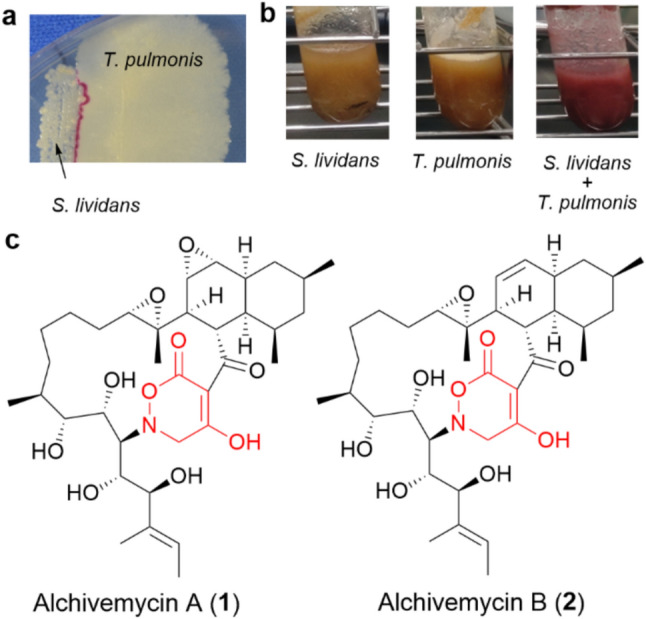
**a** Production of pigmented antibiotics in *Streptomyces lividans* TK23 (*S. lividans*) was induced by co-culturing with *Tsukamurella pulmonis* TP-B0596 (*T. pulmonis*) on an agar plate. **b** Production of pigmented antibiotics in *S. lividans* was also induced in liquid culture through co-cultivation with *T. pulmonis*. **c** Chemical structures of alchivemycins (**1** and **2**), with the 2*H*-tetrahydro-4,6-dioxo-1,2-oxazine moiety highlighted in red

Initially, they conducted a screening of bacteria capable of activating actinobacterial SM-BGCs using *Streptomyces lividans* TK23 as an indicator, which produces pigmented antibiotics (actinorhodin and undecylprodigiosin) in response to specific stimulation. As a result, *Tsukamurella pulmonis* TP-B0596 (*T. pulmonis*) was identified as an effective inducer of pigmented antibiotics production in *S. lividans* TK23, both on agar plates (Fig. 1a) and in liquid cultures (Fig. 1b) [21].

The genus *Tsukamurella* belongs to MACB, a group of bacteria characterized by the presence of mycolic acid on their cell surface. Notably, other MACB genera, such as *Corynebacterium*, *Dietzia*, and *Rhodococcus*, also induced pigment production in *S. lividans* TK23. The importance of mycolic acid was further supported by the finding that *Corynebacterium glutamicum* Δ*pks13*, a mutant lacking the mycolic acid biosynthetic gene, lost its inducing activity [21].

More importantly, MACB not only induced pigment production in *S. lividans* TK23 but also broadly activated SM-BGCs in various actinomycetes. Onaka et al. reported that co-culturing *T. pulmonis* with 112 strains of soil-derived *Streptomyces* resulted in metabolic profile changes in 97 strains, among which 35 strains exhibited enhanced secondary metabolite production [21]. In particular, two novel polyketides featuring a unique 2*H*-tetrahydro-4,6-dioxo-1,2-oxazine moiety, named alchivemycins A (**1**) and B (**2**), were isolated from *Streptomyces* sp. TP-A0867 when co-cultured with *T. pulmonis* (Fig. 1c) [21–23]. Alchivemycins exhibited potent and selective antibacterial activity against *Kocuria rhizophila* ATCC 9341 (formerly *Micrococcus luteus* ATCC 9341), with MIC values of 0.03 µg/ml for **1** and 0.004 µg/ml for **2** [23].

### Application of combined-culture strategy to *Streptomyces* strains

**Fig. 2:**
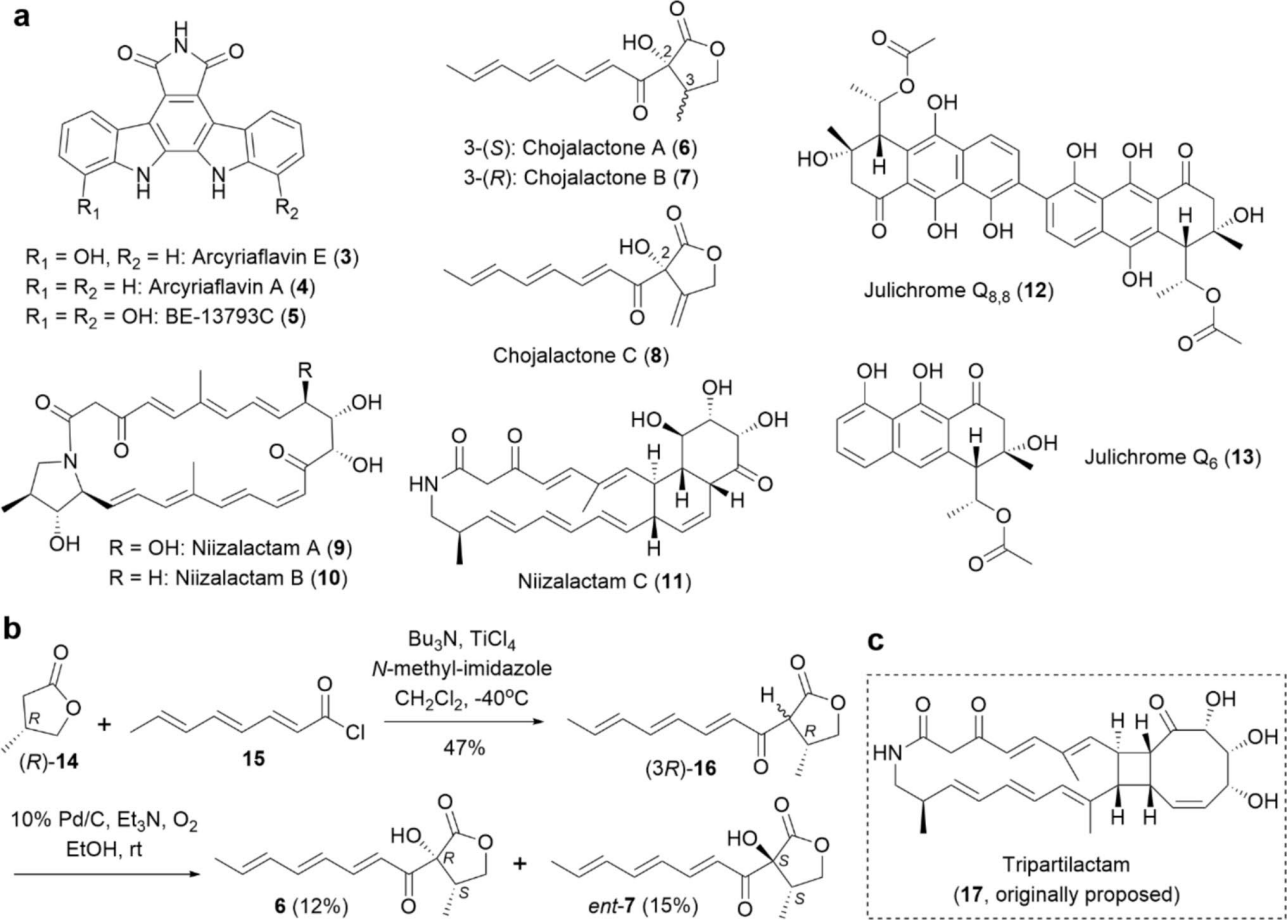
**a** Chemical structures of secondary metabolites (**3**–**13**) obtained from *Streptomyces* strains through our combined-culture screening. **b** Chemical synthesis of possible stereoisomers of **6** and **7**. The figure illustrates the case in which (*R*)-**14** was used as the starting material. **c** Chemical structure of tripartilactam (**17**), as originally proposed by Oh et al. in 2012 [33]

Encouraged by the discovery of alchivemycins, which possess unique chemical structures and potent bioactivity, we aimed to obtain new secondary metabolites through the combined-culture strategy. Initially, we targeted *Streptomyces* strains, a representative genus of actinomycetes that has produced the majority of actinobacterial secondary metabolites [3, 24]. In our combined-culture screening, *T. pulmonis* was consistently used as the MACB, and changes in the metabolic profile were monitored by HPLC equipped with a diode array detector (HPLC–DAD). Thus far, we have isolated eleven secondary metabolites, including seven new compounds, from four *Streptomyces* strains as described below (**3−13**, Fig. 2a).

*Streptomyces cinnamoneus* NBRC 13823 was found to produce three pigmented metabolites, whose production was significantly enhanced in the presence of MACB [25]. Based on chromatographic purification and spectroscopic analysis, one of these metabolites was identified as a new indolocarbazole alkaloid, named arcyriaflavin E (**3**), while the remaining two were the known indolocarbazole alkaloids arcyriaflavin A (**4**) and BE-13793C (**5**) [26, 27]. **3** and **5** exhibited moderate cytotoxicity against P388 murine leukemia cells, with IC_50_ values of 39 and 33 µM, respectively, while **4** showed no cytotoxicity up to 100 µM [25].

*Streptomyces* sp. CJ-5, which we isolated from a soil sample collected in Isumi City (Chiba, Japan), produced three unknown metabolites exclusively in the presence of MACB. Our detailed spectroscopic analysis revealed that all of these metabolites are new butanolides, named chojalactones A–C (**6−8**) [28]. The relative configurations of **6** and **7** were determined by NMR analysis, while their absolute configurations were established through the chemical synthesis of all possible enantiomers from 3-methyl-γ-butyrolactone (**14**) (Fig. 2b).

Initially, **14** was condensed with triene acid chloride (**15**) via Ti-crossed Claisen condensation using *N*-methylimidazole as a catalyst [29], yielding the β-ketolactone (**16**). **16** was then subjected to Pd/C-catalyzed direct α-oxidization under an O_2_ atmosphere [30] to obtain the desired stereoisomers: (2*R*, 3*S*) and (2*S*, 3*S*) enantiomers from (3*R*)-**16**, and (2*S*, 3*R*) and (2*R*, 3*R*) enantiomers from (3*S*)-**16**. By comparing the retention times of the natural products with those of synthetic standards using chiral-phase HPLC, the absolute configurations of **6** and **7** were determined to be (2*R*, 3*S*) and (2*R*, 3*R*), respectively. The absolute configuration at the C-2 position of **8** was deduced to be *R* based on its consistency with **6** and **7**. Finally, **6** and **7** exhibited moderate cytotoxicity against P388 murine leukemia cells, with IC_50_ values of 28 and 18 µM, respectively.

It was found that *Streptomyces* sp. NZ-6, which we isolated from a soil sample collected in Niiza City (Saitama, Japan), produces three metabolites in a MACB-dependent manner. Two of them were identified as new polyene macrolactams with a common [5, 23]-bicyclic skeleton and were named niizalactams A (**9**) and B (**10**) [31]. The relative and absolute configurations of **9** and **10** were fully determined based on exhaustive NMR analysis of the natural products and their synthetic derivatives, including the application of the modified Mosher’s method to secondary alcohols [32].

The NMR spectrum of the remaining metabolite closely matched that of tripartilactam (**17**), a polyene macrolactam with an [4, 8, 18]-tricyclic skeleton, which was isolated from *Streptomyces* sp. SNA112 by Oh et al. in 2012 (Fig. 2c) [33]. However, based on our thorough NMR analysis, the presence of an [6, 6, 18]-tricyclic skeleton was supported, leading to its designation as niizalactam C (**11**). Recently, Oh et al*.* conducted a re-investigation of the chemical structure of **17**, including a ^13^C–^13^C COSY analysis of a ^13^C-labeled derivative of **17** [34]. In their study, the original structure of **17**, proposed in 2012, was revised to be identical to **11**, as proposed by our group.

Along with the above examples, we also found that *Streptomyces* sp. TAKO-2, which we isolated from a soil sample collected in Tako Town (Chiba, Japan), produced compounds with absorption in the long-wavelength region (> 400 nm) when co-cultured with MACB [35]. The spectral data of two of these metabolites matched those of the known aromatic polyketides, julichrome Q_8,8_ (**12**) [36] and julichrome Q_6_ (**13**) [37]. Considering the similarity of their UV–vis spectra, the remaining metabolites were also inferred to be related analogs to **12** and **13**.

### Application of combined-culture screening to non-*Streptomyces* actinomycetes

**Fig. 3:**
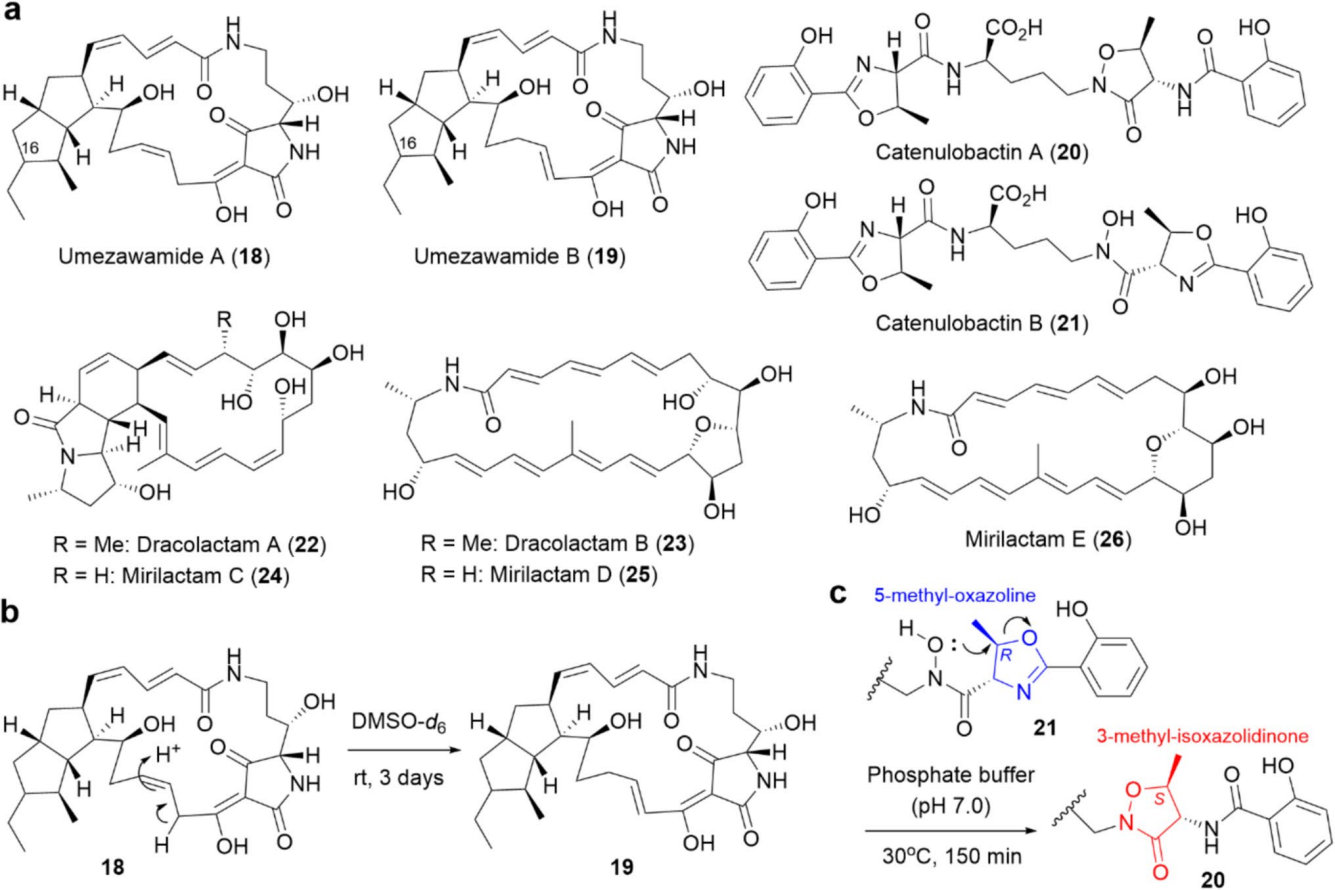
**a** Chemical structures of secondary metabolites (**18**–**26**) obtained from non-*Streptomyces* strains through our combined-culture screening. **b 18** spontaneously converts to **19** in DMSO-*d*_*6*_ solution, accompanied by double bond isomerization. **c**. Non-enzymatic isomerization of **21**, leading to the formation of the 3-methyl-isoxazolidinone scaffold in **20**

As describe above, our combined-culture screening initially targeted *Streptomyces* strains; however, we later shifted our focus to other actinomycete genera. Non-*Streptomyces* actinomycetes, often referred to as rare actinomycetes, have been relatively unexplored for secondary metabolites compared to *Streptomyces*. However, recent genomic studies of non-*Streptomyces* genera have revealed the presence of SM-BGCs comparable to or even exceeding those in *Streptomyces* [7, 8]. In fact, a considerable number of new bioactive secondary metabolites have been identified from these genera [5, 38]. Thus far, through combined-culture screening, we have identified nine new secondary metabolites from four non-*Streptomyces* actinomycetes (**18**–**26**, Fig. 3a).

The genus *Umezawaea* is a relatively new actinomycete genus proposed in 2007 [39], and no reports on its secondary metabolites had been published until we started the combined-culture screening. *Umezawaea* sp. RD066910 does not produce any notable metabolites under pure-culture conditions; however, we found that it produced two metabolites with similar UV spectra when co-cultured with MACB. Spectral analysis of the purified metabolites revealed that both are new polycyclic tetramate macrolactams and represent the first secondary metabolites identified from the genus *Umezawaea*. We named them umezawamides A (**18**) and B (**19**), which are structural isomers differing only in the position of the carbon–carbon double bonds [40].

During NMR analysis, it was found that **18** gradually converted to **19** in DMSO-*d*_6_ at room temperature, suggesting that **19** is an artifact generated from **18** (Fig. 3b). The absolute configurations of **18** and **19**, except at the C-16 position, were fully elucidated by combining NMR analysis, conformational search using the Merck Molecular Force Field (MMFF), and ECD calculations. **18** and **19** exhibited cytotoxicity against P388 murine leukemia cells, with IC_50_ values of 3.7 and 4.8 µM, respectively. Additionally, **18** showed antifungal activity against *Candida albicans*, whereas **19** did not [40].

*Catenuloplanes* sp. RD067331 exhibited a significant increase in the production of two metabolites when cultured in the presence of MACB. Spectral analysis revealed that these metabolites are new heterocyclic peptides, which we named catenulobactins A (**20**) and B (**21**) [41]. Total acid hydrolysis, followed by chiral-phase GC–MS analysis of **21**, revealed that *N*^5^-hydroxyornithine is in the d-form, and that both 5-methyl-oxazolines (MeOzn) are derived from l-threonine, leading to the complete determination of its absolute configuration.

**20** is a structural isomer of **21**, featuring a unique 3-methyl-isoxazolidinone ring in place of one of the MeOzn moieties. Considering the biosynthetic mechanism of acinetobactin proposed by Wencewicz et al. [42], the 3-methyl-isoxazolidinone ring in **20** is presumed to be generated through an intramolecular rearrangement of **21**, involving the MeOzn moiety and the adjacent *N*–OH group (Fig. 3c). Notably, we found that **21** spontaneously converted to **20** when incubated in phosphate buffer (pH 7.0) at room temperature [41]. Finally, we found that **21** exhibits Fe(III)-chelating activity as well as cytotoxicity against P388 murine leukemia cells, with an IC_50_ value of 22.4 µM, whereas **20** exhibited neither activity [41].

*Micromonospora wenchangensis* HEK-797, isolated from lake sediment at Hegura Island (Ishikawa, Japan), produced two metabolites specifically in the presence of MACB. NMR analysis of the purified metabolites revealed that one possesses a unique [5, 5, 6, 16]-tetracyclic skeleton, which we named dracolactam A (**22**), while the other is a new bicyclic lactam featuring a THF ring, named dracolactam B (**23**) [43]. The relative and absolute configurations of **22** and **23** were determined through NOESY analysis and a careful evaluation of vicinal coupling constants of the natural products, combined with detailed NMR analysis of the corresponding acetonide derivatives and the application of the modified Mosher’s method.

### Epoxidation-mediated intramolecular cyclization of polyene macrolactams triggered by combined-culture

**Fig. 4:**
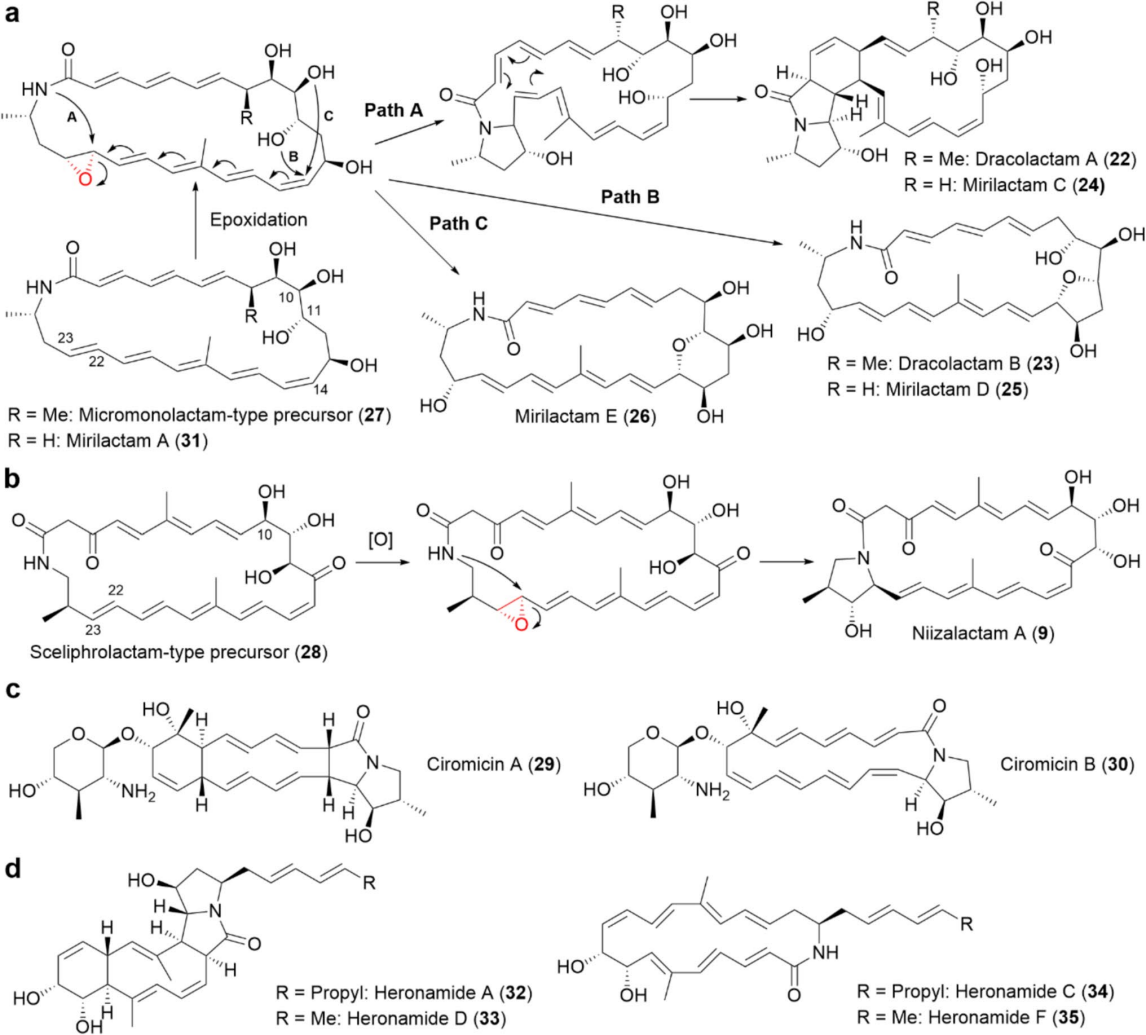
**a** Proposed biosynthetic pathways of dracolactams (**22** and **23**) and mirilactams C–E (**24**–**26**), involving epoxidation-mediated intramolecular cyclization of 26-membered macrolactam precursors. **b** Proposed biosynthetic pathway of niizalactam A (**9**) from a 26-membered sceliphrolactam-type precursor (**28**). Niizalactam B (**10**) is also presumed to be biosynthesized from the C-10 deoxy derivative of **28**. **c** Chemical structures of ciromicins A (**29**) and B (**30**). **d** Chemical structures of heronamides (**32**–**35**)

Considering the positions of functional groups, **22** and **23** were presumed to originate from a common 26-membered polyene macrolactam (**27**), whose planar structure is identical to those of micromonolactam [44] and isomicromonolactam [45] (Fig. 4a). In our proposed mechanism, the double bond between C-22 and C-23 of **27** is initially epoxidized, generating an unstable intermediate, which subsequently undergoes intramolecular cyclization.

If the amide nitrogen directly attacks the epoxide ring at C-22, the resulting bicyclic compound undergoes further intramolecular Diels–Alder reaction, forming **22** (Fig. 4a, Path A). Meanwhile, if the hydroxyl group at C-11 attacks the C-14 carbon, inducing a 1,10-addition accompanied by epoxide ring opening, **23** is formed (Fig. 4a, Path B). Notably, *M. wenchangensis* HEK-797 produced a metabolite with a UV–Vis spectrum and molecular formula identical to **27** under both pure-culture and combined-culture conditions, suggesting that MACB specifically activates the epoxidation of **27** rather than its biosynthesis [43].

A similar biosynthetic strategy can also be applied to **9** and **10**, in which a 26-membered polyene macrolactam (**28**), sharing the same planar structure as sceliphrolactam [46], or its C-10 deoxy derivative, is presumed to be the precursor (Fig. 4b). It was assumed that epoxidation at the C-22 and C-23 double bond, followed by nucleophilic pyrrolidinol formation, leads to the generation of **9** and **10** (Fig. 4b). Additionally, Bachmann et al. reported that the rare actinomycete *Nocardiopsis* sp. FU40 ΔApoS produced ciromicins A (**29**) and B (**30**) (Fig. 4c) strictly in the presence of MACB (*Rhodococcus wratislaviensis*) [47]. **29** and **30**, which contain the pyrrolidinol moiety, are proposed to be biosynthesized through the epoxidation of a 22-membered glycosylated polyene macrolactam, followed by intramolecular cyclization [47].

Based on these findings, we hypothesized that applying combined-culture could activate cryptic epoxidation-mediated cyclization pathways in polyene macrolactam-producing actinomycetes. To validate this hypothesis, we focused on *Actinosynnema mirum* NBRC 14064 (*A. mirum*), which is known to produce a 26-membered polyene macrolactam named mirilactam A (**31**) (Fig. 4a) [48].

We confirmed that *A. mirum* produces **31** under monoculture conditions, and as expected, specifically produced three additional metabolites when co-cultured with MACB [49]. Detailed structural analysis of the purified metabolites revealed that they are all new compounds derived from **31**, which we named mirilactams C–E (**24**−**26**, Fig. 3a) [49]. The biosynthetic mechanism of **24** and **25** is similar to that of **22** and **23**. Meanwhile, in the biosynthesis of **26**, it is likely that the hydroxyl group at C-10 in the epoxidized intermediate attacks the C-14 carbon, leading to the formation of a tetrahydropyran ring (Fig. 4a, Path C).

Polycyclic metabolites, likely generated by the epoxidation of polyene macrolactams, have also been found in the mono-culture of actinomycetes. Heronamide A (**32**) is a [5, 5, 6, 10]-tetracyclic metabolite containing a pyrrolidinol moiety, identified from the marine-derived *Streptomyces* sp. CMB-M0406 (Fig. 4d) [50, 51], while heronamide D (**33**), which shares the same tetracyclic skeleton as **32**, was identified from the deep-sea-derived *Streptomyces* sp. SCSIO 03032 (Fig. 4d) [52].

Strains CMB-M0406 and SCSIO 03032 also produce 20-membered polyene macrolactams, named heronamides C (**34**) and F (**35**), respectively [50, 52, 53] (Fig. 4d), which are likely precursors of **32** and **33**. Notably, the production of **32** significantly increased when artificial seawater was added to the culture medium, whereas no difference was observed in the production of **34** between saline and non-saline conditions [50]. Additionally, the production of **33** was observed in the medium containing artificial seawater [52], suggesting that salt stress induce the epoxidation of polyene macrolactams in certain actinomycetes.

To the best of my knowledge, no epoxidase genes have been clearly identified within the polyene macrolactam BGCs. Thus, it is possible that an epoxidase gene located outside the polyene macrolactam BGC is activated during co-cultivation with MACB. On the other hand, it has been reported that **34** undergoes a non-enzymatic conversion to **32** in DMSO at room temperature [53], raising the possibility that the MACB-induced epoxidation could occur through a non-enzymatic mechanism, such as the generation of reactive oxygen species.

### Identification and biosynthetic studies of bisenarsan: a novel organoarsenic secondary metabolite produced by model actinomycetes

Organoarsenic compounds containing C–As bonds are quite rare among bacterial natural products. A representative example is a series of simple metabolites biosynthesized through the sequential methylation of arsenite [As(III)], often followed by oxidation, a process widely recognized as a detoxification mechanism for highly toxic inorganic arsenic [54–56]. In other examples, several cyanobacteria have been reported to produce oxo-arsenosugars [57, 58], while a potent glutamine synthetase inhibitor, arsinothricin, has been identified from the terrestrial bacterium *Burkholderia gladioli* GSRB05 [59, 60].

In the biosynthesis of these organoarsenic natural products in bacteria, all C–As bonds are formed by *S*-adenosylmethionine (SAM)-dependent enzymes, among which SAM-dependent arsenic methyltransferases are widely distributed in bacterial genomes [54–56]. In addition, recent studies have shown that radical SAM enzymes are also involved in C–As bond formation. The adenosylation of arsenic in oxo-arsenosugar biosynthesis [61] and the 3-amino-3-carboxypropyl radical transfer to the arsenic atom in arsinothricin biosynthesis [62] are both catalyzed by radical SAM enzymes.

**Fig. 5:**
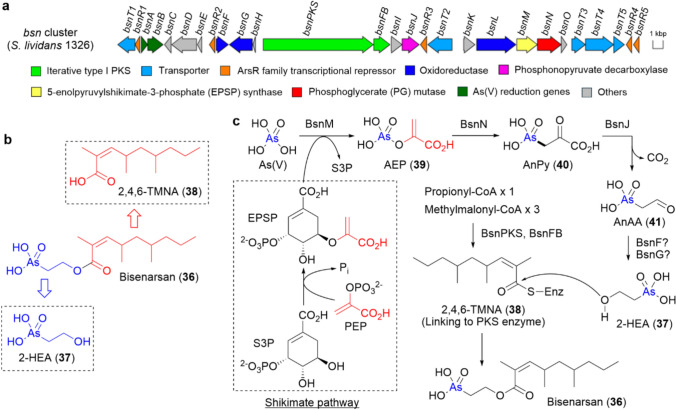
**a** Schematic representation of the SM-BGC for **36** in *Streptomyces lividans* 1326 (*bsn* cluster). **b** Chemical structure of **36**, along with its proposed components (**37** and **38**). **c** Proposed biosynthetic pathway of **36** from As(V)

Despite their high capacity for secondary metabolite production, actinomycetes have long been known to produce only simple methylated arsenicals [56]. However, in 2016, Cruz-Morales et al. reported that the two model actinomycetes *Streptomyces lividans* 1326 and *Streptomyces coelicolor* A3(2) produce an unknown organoarsenic metabolite (**36**, C_14_H_27_AsO_5_) [63]. They further proposed that a conserved SM-BGC among these strains, which we later termed the *bsn* cluster, is responsible for the biosynthesis of **36** (Fig. 5a) [63].

The *bsn* cluster includes genes involved in arsenic resistance and arsenic-dependent transcriptional regulation. In fact, it has been reported that the transcription of several genes within the *bsn* cluster is significantly upregulated under exposure to arsenate [As(V)] [63]. More importantly, the *bsn* cluster does not contain any genes encoding SAM-dependent enzymes. However, it has been reported that disruption of the 5-enolpyruvylshikimate-3-phosphate (EPSP) synthase homolog (*bsnM*) completely abolished the production of **36** [63].

Based on these findings, **36** was expected to be an organoarsenic natural product biosynthesized through C–As bond formation without the involvement of SAM-dependent enzymes. Therefore, we aimed to determine the chemical structure of **36**. Initially, *S. lividans* 1326 was mass-cultured in a medium containing As(V) as the sole arsenic source, but only a trace amount of **36** (< 0.1 mg) was obtained [64]. However, ^1^H NMR and HR-MS/MS analysis of the trace purified material suggested that **36** is an *O*-acyl derivative of (2-hydroxyethyl)arsonic acid (2-HEA, **37**) (Fig. 5b).

In addition, Wang et al. reported that heterologous expression of the polyketide synthase (PKS) genes (*bsnPKS* and *bsnFB*, Fig. 5a) resulted in the production of 2,4,6-trimethyl-2-nonenoic acid (2,4,6-TMNA, **38**) [65], suggesting that the acyl group of **36** is derived from **38** (Fig. 5b). Notably, when chemically synthesized **37** was added to the culture medium, the production of **36** in *S. lividans* 1326 significantly increased. Finally, NMR re-analysis of the purified metabolite confirmed that the chemical structure of **36** matched our initial proposal, and we named it bisenarsan (Fig. 5b) [64].

Through gene disruption in *S. lividans* 1326, we revealed that, in addition to *bsnM* (EPSP synthase) [63], genes encoding phosphoglycerate mutase (*bsnN*) and phosphonopyruvate decarboxylase (*bsnJ*) are also required for the biosynthesis of **36** (Fig. 5a) [64]. Furthermore, supplementation with chemically synthesized **37** in the culture medium of all the disruptants of *bsnM*, *bsnN*, and *bsnJ* restored the production of **36**, suggesting that these genes are responsible for the biosynthesis of **37** [64].

Based on these observation as well as the predicted functions of *bsn* genes, we propose the biosynthetic pathway of **36** as follows (Fig. 5c). Initially, the pyruvate group in EPSP, presumably derived from the shikimate pathway, is transferred to As(V) by BsnM, resulting in the formation of arsenoenolpyruvate (AEP, **39**). **39** is then converted to arsonopyruvate (AnPy, **40**) by BsnN, accompanied by C–As bond formation, and subsequently decarboxylated to arsonoacetoaldehyde (AnAA, **41**) by BsnJ. **41** is presumed to be reduced to **37** by reductases encoded within the *bsn* cluster or by an orphan reductase in *S. lividans* 1326. Finally, **38**, bound to the acyl carrier protein of the PKS module (BsnPKS and BsnFB), is thought to undergo nucleophilic attack by **37**, leading to the release of **36**. It has been reported that the expression of *bsnPKS* is upregulated upon exposure to As(V) [63], suggesting that ester bond formation between **37** and **38** is part of the genuine biosynthetic pathway rather than a shunt pathway.

The biological function of **36** remains unclear; however, we have previously shown that its toxicity is significantly lower than that of As(V) [64], suggesting that the biosynthesis of **36** may serve as a detoxification mechanism for inorganic arsenic. Additionally, we have demonstrated that **37** exhibits stronger antibacterial activity against *Staphylococcus aureus* than As(V) [64], raising the possibility that **36** is produced as “Trojan horse” antibiotic, with **37** serving as the active form.

Intriguingly, the gene set *bsnMN*, responsible for the conversion of inorganic arsenic [As(V)] to **40**, is widely distributed among actinobacterial genomes, whereas the surrounding regions exhibit considerable genetic diversity [63, 64].

## Conclusion

As described earlier, we identified a total of 20 actinobacterial secondary metabolites, including 16 new compounds, from *Streptomyces* strains (**3−13**, Fig. 2a) and non-*Streptomyces* genera (**18−26**, Fig. 3a) by activating cryptic SM-BGCs through co-culture with MACB (combined-culture). The continued application of the combined-culture strategy to other actinomycetes is expected to facilitate the discovery of additional new bioactive natural products. In fact, the number of new secondary metabolites obtained through this strategy has continued to increase in recent years [66–69].

Furthermore, we isolated and determined the chemical structure of a novel organoarsenic metabolite, named bisenarsan (**36**), produced by two model actinomycetes (Fig. 5b). Additionally, our study provided significant insight into the biosynthesis of **36**, suggesting that C–As bond in **36** is likely generated through a novel pathway that does not involve SAM-dependent enzymes (Fig. 5c). Notably, the *bsnMN* homologs, presumably responsible for the generation of AnPy (**40**) from As(V), are widely distributed among actinobacterial genomes, and genome mining targeting these homologs is expected to facilitate the discovery of new organoarsenic metabolites that share **40** as a common precursor.

Overall, a large number of untapped secondary metabolic pathways still exist in actinomycetes, and we continue to explore and utilize these pathways to discover new bioactive secondary metabolites.
